# The impact of physical play-based games on executive functions and social behaviors in children with autism spectrum disorder: a systematic review and meta-analysis

**DOI:** 10.3389/fpsyt.2026.1782760

**Published:** 2026-03-23

**Authors:** Mengyao Feng, Shenning Zhou, Yaoqi Hou, Xiangqin Song

**Affiliations:** School of Physical Education and Sports, Beijing Normal University, Beijing, China

**Keywords:** children with autism spectrum disorder, executive function, meta-analysis, physical play-based games, social behavior

## Abstract

**Objective:**

To conduct a systematic review and meta-analysis examines the impact of physical play-based games on executive functions and social behaviors in children with Autism Spectrum Disorder (ASD),and to compare the differences in effects across intervention types and dosages through subgroup analysis. To our knowledge, this is the first meta-analysis to concurrently evaluate these two domains in this population.

**Methods:**

Six databases (CNKI, Wanfang, PubMed, Cochrane Library, Web of Science, Embase) were searched from inception to December 20, 2025, for randomized controlled trials (RCTs) and non-RCTs on physical play-based Games interventions for children with ASD. Two researchers independently screened studies, extracted data, and assessed using the Cochrane Risk of Bias tool (RoB 2) for randomized controlled trials and the ROBINS-I tool for non-randomized studies. Meta-analysis was performed using STATA 18.0 with a random-effects model. Effect sizes were expressed as standardized mean differences (SMD) with 95% confidence intervals (CI).

**Results:**

Twelve studies involving 520 children with ASD were included. Meta-analysis showed that physical play-based games significantly improved social behavior in children with ASD (SMD = 0.68, 95% CI [0.49, 0.88], P < 0.001). However, the improvement in executive function did not reach statistical significance (SMD ≈ 0.31, 95% CI [-0.04, 0.67], P > 0.05). Given the limited number of studies (n=4) and the marginal nature of this finding, this result should be interpreted with caution and requires confirmation in future research. Subgroup analysis indicated that traditional physical play-based games, high-frequency (≥4 sessions/week), and longer-duration (≥60 minutes/session) interventions showed numerically larger effect sizes, but the differences between subgroups were not statistically significant.

**Conclusion:**

physical play-based games are an effective intervention for improving social behavior in children with ASD, with a moderate and consistent effect. Structured group-based physical play-based games are recommended in practice, with adjustments based on individual child characteristics. Further high-quality research is needed to verify long-term effects and generalization.

## 1 Introduction

Autism spectrum disorder (ASD) is a common, highly heritable, and heterogeneous neurodevelopmental disorder (1). Children with ASD commonly face two core challenges: executive dysfunction and social behavioral difficulties (2, 3). These two types of impairments are interrelated and collectively affect their learning, daily living, and social adaptation abilities (4).

Executive functions (EFs) constitute a set of cognitive operations that serve as the foundation for selecting, scheduling, coordinating, and monitoring complex, goal-directed processes involving perception, memory, and action (5, 6). Children with autism spectrum disorder (ASD) exhibit significant deficits in these areas, such as difficulties in inhibiting inappropriate responses, retaining information in working memory, and flexibly switching between tasks (7). These cognitive impairments limit their ability to plan actions, solve problems, and adapt to environmental changes.

Meanwhile, children with ASD exhibit significant difficulties in social interactions, including challenges in understanding and utilizing nonverbal social cues (e.g., facial expressions, eye contact, tone of voice), initiating or maintaining conversations, and engaging in cooperative activities (8–10). Notably, executive functions are closely linked to social behavior during development: effective social interactions often require working memory to track information, inhibitory control to manage impulses, and cognitive flexibility to adapt to different social situations. Therefore, deficits in executive functions may partially explain the social behavioral difficulties in children with ASD (11), and interventions targeting these core cognitive processes may indirectly or directly enhance the social participation abilities of children with ASD.

Given the aforementioned challenges, identifying effective and feasible interventions is crucial. Among various non-pharmacological interventions, physical activity-based games demonstrate unique potential. These refer to structured or semi-structured activities that are grounded in physical movement and incorporate rules and game elements, aimed at promoting children’s motor, cognitive, and social development (12, 13). Specific forms include, but are not limited to, traditional group games (e.g., chase games, ball games), sensory games (e.g., Kinect-based activities), and other action-based recreational activities. Compared to many traditional interventions, such activities typically offer advantages such as high engagement, flexible formats, ease of implementation, and absence of side effects, and are increasingly recognized for their role in improving core symptoms in children with ASD (10, 14).

Previous studies have explored the broad benefits of physical activity for children with ASD (13).More recently, a comprehensive meta-analysis synthesized evidence from randomized controlled trials and reported significant improvements in social and motor functions following physical activity interventions in children with ASD, although the effects on executive function were less consistent (15).However, evidence specifically targeting physical activity games and systematically evaluating their impact on two key and interrelated domains—executive function and social behavior—remains to be integrated. Additionally, it is unclear how the specific characteristics of interventions (e.g., type, frequency, duration) modulate their effects. Therefore, this study aims to comprehensively assess the intervention effects of physical activity games on executive function and social behavior in ASD children through systematic reviews and meta-analyses. Furthermore, subgroup analyses will be conducted to explore the moderating role of intervention type and dosage (e.g., frequency, duration), thereby providing more targeted evidence-based insights for clinical practice.

## 2 Methods

This review and meta-analysis comply with the 2020 updated PRISMA (Preferred Reporting Practice for Systematic Reviews and Meta-Analyses) guidelines, with the PRISMA checklist available in the **Supplementary Materials** (Registration Number: CRD420251274556).

### 2.1 Literature search strategy and process

A comprehensive search was conducted on the topic of “the impact of physical play-based games on the executive functions and social behaviors of children with Autism Spectrum Disorder” to identify all studies reporting changes in EFs and social behaviors following physical play-based game interventions for children with autism. Six databases were systematically searched: China National Knowledge Infrastructure (CNKI), WanFang, PubMed, Cochrane Library, Web of Science, and Embase, from their inception to December 20, 2025.

The search strategy employed a combination of keywords and Medical Subject Headings (MeSH) terms, constructed around four core concepts: Autism Spectrum Disorder (autism, ASD, etc.), Physical Play-based Games (physical games, physical exercise, etc.), Executive Functions (cognitive control, inhibitory control, working memory, etc.), Social Behaviors (social skills, social interaction, etc.), and Children (child, preschool, etc.). Synonyms within each conceptual group were combined using the Boolean operator “OR”, and the different conceptual groups were combined using “AND”. To ensure complete transparency and reproducibility of the research process, the reproducible search syntax executed across all six databases is provided in the appendix. The following is an example of the core keyword framework upon which the search syntax was built. The reproducible search syntax executed across all six databases is provided in the **Appendix**. The detailed search strings for each database are also provided in **Supplementary Table 1**. **Table 1** presents an example of the core keyword framework used to construct the search query.

**Table 1:** The core concepts and keyword framework of the search strategy.

Core concept	English key words
Person	“Autism Spectrum Disorder”, autistic, ASD, asperger*
Intervention	“physical play-based Games”, “Physical Play”, Exercise, “Physical Activity”, Sport*, “Motor Intervention”, “Play-Based Intervention”, “Recreational Therapy”
Outcome 1	“Executive Function”, Cognitive Control, Inhibition, “Working Memory”, Cognitive Flexibility, Planning, Attention Control
Outcome 2	“Social Behavior”, “Social Skills”, “Social Interaction”, “Social Communication”, “Social Responsiveness”, “Peer Interaction”
Range	child, preschool, kid, toddler*

**Figure 1:**
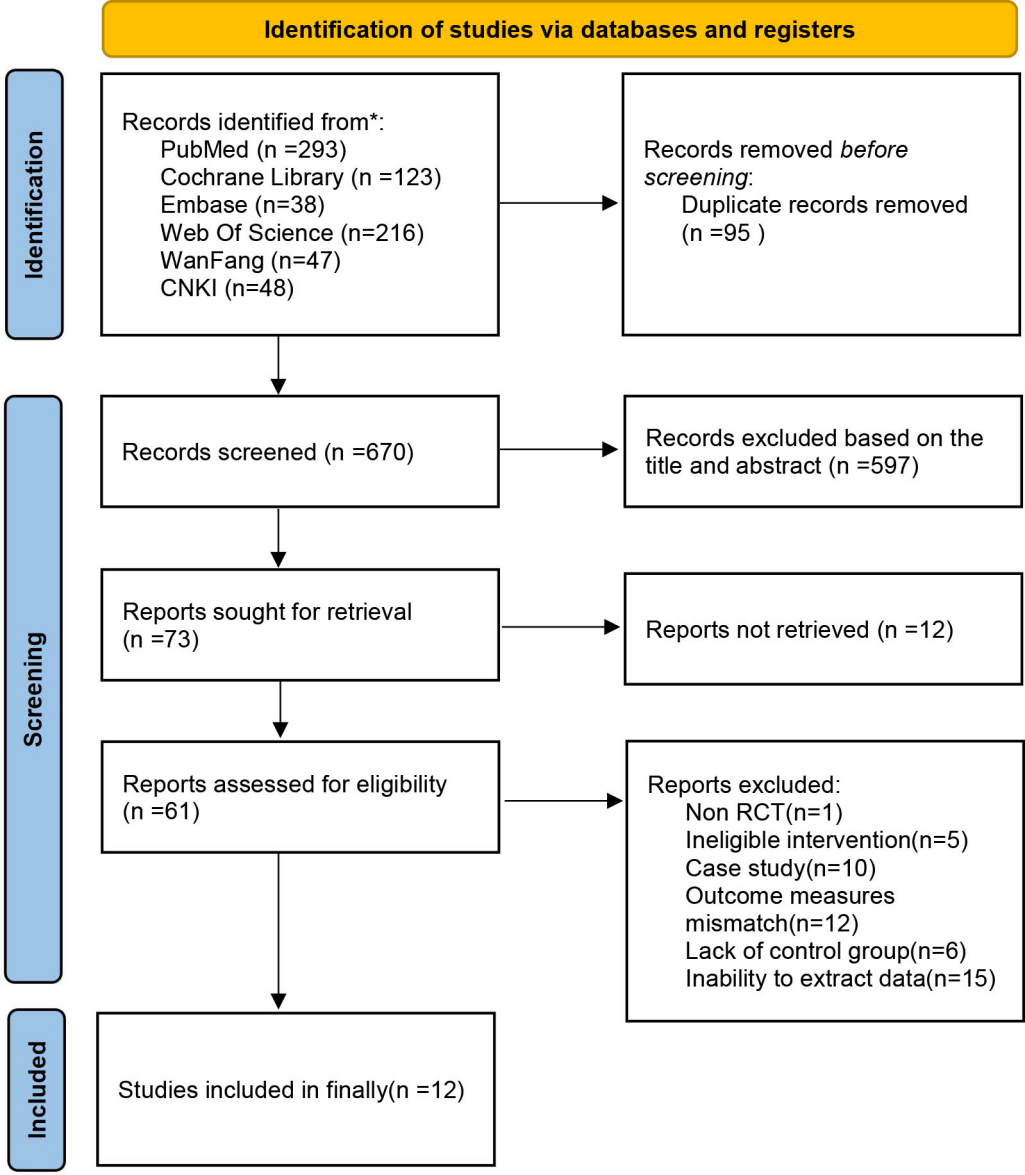
Flow diagram of study selection.

**Figure 1** illustrates the study selection process. The initial search across six literature databases yielded a total of 765 records. Following the removal of duplicate records, the full texts of the remaining 670 records were assessed. Of these, 658 articles were excluded for the following reasons: excluded based on title and abstract (n = 386), non-RCT (n = 1), intervention type did not match (n = 5), case study (n = 10), outcome measures did not match (n = 12), lack of a control group (n = 6), inability to extract data (n = 15), and unavailability of full text (n = 12). Consequently, 12 studies were ultimately included in the systematic review.

### 2.2 Inclusion and exclusion criteria

Literature retrieved from the searches was screened using Zotero software. Inclusion criteria were established according to the PICOS principle. The study population (P) comprised children aged 3–12 years with a confirmed diagnosis of Autism Spectrum Disorder. The intervention (I) was physical play-based games, including but not limited to: physical games, exercise intervention, physical training, physical activity, sports, game-based intervention, recreational therapy, and Kinect-based activities. The comparator (C) was a blank control, regular activity, or a different type of exercise intervention from that used in the experimental group. Outcome measures (O) were scores from relevant assessment tools, such as the ATEC, ABC, CARS, SRS, SRS-2, SSBS-2, MABC-2, HAAR, PEDI, and WCST, which measure executive functions and social behaviors. Study design (S) included randomized controlled trials (RCTs) and non-randomized controlled trials.

Exclusion criteria were as follows: ① Study population comprised non-ASD individuals or individuals with ASD comorbid with other severe neurological/musculoskeletal diseases; ② The intervention was not a physical play-based game, or the study design did not allow for a comparison highlighting the value of the physical play-based game intervention (e.g., no clear contrast between groups); ③ Literature not published in Chinese or English; ④ Literature for which the full text was unavailable, data were incomplete, or was a duplicate publication; ⑤ Studies that did not report complete data for the assessment tools relevant to the executive functions and social behaviors of interest in this review; ⑥ Study design was a non-controlled trial.

### 2.3 Outcome measures and assessment tools

The primary outcome measures of this study were changes in scores on the Wisconsin Card Sorting Test (WCST) for executive function and changes in standardized assessment scores for social behavior in children with ASD. The included studies employed a variety of internationally recognized assessment tools, mainly including: (1) Executive Function Tests: The Wisconsin Card Sorting Test (WCST), the Movement Assessment Battery for Children – Second Edition (MABC-2), and the Pediatric Evaluation of Disability Inventory (PEDI, PEDI-CAT). These tools provide composite or subscale scores for dimensions such as executive function and daily social skills. (2) Social Behavior Tests: The Autism Treatment Evaluation Checklist (ATEC), the Autism Behavior Checklist (ABC), the Childhood Autism Rating Scale (CARS), the Social Responsiveness Scale and its Second Edition (SRS, SRS-2), and the Social Skills Rating System – Second Edition (SSBS-2), which are specifically designed to assess social behavior. (3) Other Tools: The Hong Kong Assessment of Motor Abilities of Children with Autism (HAAR), etc.

Despite the diversity of tools, their assessment cores revolve around the two key domains of executive functions and social behavior. For data synthesis, we extracted the post-test total scores, standard deviations, and sample sizes for the standardized assessment tools targeting these two outcome measures from each study. The total scores from all included tools were considered valid measures of overall function in their respective domains and were directly used in the meta-analysis. If a single study used multiple tools to assess the same construct (e.g., using both SRS and CARS to assess social behavior), we extracted the total score data for each tool separately and treated them as independent data points in the analysis.

While tools such as the Movement Assessment Battery for Children – Second Edition (MABC-2) and the Pediatric Evaluation of Disability Inventory (PEDI) primarily assess motor skills and daily functioning, some subscales or items may reflect aspects of executive function (e.g., planning, organization) in real−world contexts; however, they are not considered direct measures of core executive functions. Therefore, the interpretation of results obtained with these instruments should be made with caution.

### 2.4 Literature screening and data extraction

Two reviewer independently screened the titles and abstracts of all records retrieved from the database searches using Zotero. Full texts of potentially eligible articles were then retrieved and independently assessed against the predefined inclusion criteria. Any disagreements were resolved through discussion or, if necessary, consultation with a third reviewer. Data extraction was performed independently by the same two reviewers using a standardized data extraction form. The following information was extracted from each included study: first author and year, country, participant characteristics (sample size, age range, diagnostic criteria), intervention details (type, frequency, duration, setting), comparator, outcome measures (tools for executive function and social behavior), and main findings. In cases of missing data, we contacted the corresponding authors via email.

### 2.5 Methodological quality assessment

Two researchers independently assessed the methodological quality of the included randomized controlled trials and quasi-experimental studies using the Cochrane Risk of Bias tool. The assessment focused on: random sequence generation, allocation concealment, blinding of participants and personnel, completeness of outcome data, selective reporting, and other potential biases. Any disagreements in quality ratings were resolved through discussion to reach a consensus, involving a third researcher when necessary (16).

### 2.6 Effect size and statistical model

Data synthesis and analysis were performed using the STATA (version 18.0) software meta-analysis commands (17). For continuous outcomes, as the included studies used different standardized tools with varying scales and units to assess core indicators like EFs and social behavior, the Standardized Mean Difference (SMD) was employed as the pooled effect size to eliminate scale differences between tools and enable data combination. The SMD was calculated based on Hedges’ *g* to correct for small sample bias. The SMD is standardized by the internal standard deviation of the effect size, reflecting the degree of intervention effect relative to within-group variation. Fully acknowledging the potential heterogeneity arising from different tools and studies, a random-effects model was used for pooling to provide a more conservative estimate of the pooled effect size and its confidence interval. In result interpretation, a positive SMD indicates that the physical play-based game intervention group performed better than the control group on the executive function or social behavior measure. According to Cohen’s conventional criteria, an SMD ≈ 0.2 is considered a small effect, 0.5 a medium effect, and 0.8 a large effect.

### 2.7 Assessment of heterogeneity

The I² statistic was used to assess heterogeneity between studies. In this study, I² values of 25%, 50%, and 75% were interpreted as indicating low, moderate, and high heterogeneity, respectively. If I² > 50%, it was considered that non-negligible heterogeneity existed, and analysis of study characteristics would be conducted to explore potential sources.

### 2.8 Subgroup analysis and sensitivity analysis

To explore sources of heterogeneity and analyze the impact of intervention characteristics in depth, pre-specified subgroup analyses were conducted for the social behavior outcome measure. Analyses were primarily categorized based on the following intervention features:

Intervention Type: Based on the core format and content, divided into (1) Traditional Physical Play-based Game Intervention, referring to conventional movement games based on physical activity with fixed rules and structure; (2) Combined Intervention, referring to integrated programs that combine physical play-based games with other therapeutic methods (e.g., sensory integration training, cognitive-behavioral strategies, or direct social skills instruction).

Intervention Duration per Session: Based on the length of a single session, divided into subgroups of ≤60 minutes/session and >60 minutes/session, to examine the potential impact of single-session intensity. This classification is based on theories of attentional load and fatigue thresholds (18). Sixty minutes is a common unit length for many educational and rehabilitation sessions; using this threshold helps explore whether there exists an optimal “duration window per session” for maintaining engagement and learning efficiency.

Intervention Frequency: Based on the weekly implementation frequency, divided into subgroups of <3 sessions/week, = 3 sessions/week, and >3 sessions/week. Lower frequencies (e.g., 1–2 sessions/week) might be insufficient to counteract the generalization difficulties commonly seen in social skill acquisition among children with ASD (19); whereas very high frequencies (e.g., daily sessions) may be limited by practical feasibility (e.g., family burden, child tolerance) (20). Differentiating frequency levels allows for assessing whether a “minimum effective frequency” is needed to improve social behavior and whether higher frequencies lead to a linear increase in effects, thereby providing empirical reference for developing feasible intervention plans.

We will conduct between-group difference tests to determine whether the differences in pooled effect sizes among subgroups are statistically significant, thereby systematically examining the moderating role of the aforementioned intervention characteristics on the improvement of social behavior. Given the limited number of studies reporting executive function outcomes, this study only performed pre-specified subgroup analyses for social behavior outcomes. Since the number of studies within each subgroup is relatively small, these subgroup analyses should be considered exploratory, and their statistical power may be limited.

### 2.9 Sensitivity analysis

To test the consistency of the pooled results, sensitivity analyses were conducted as follows: (1) Excluding studies with an overall risk of bias rated as “high risk”; (2) Excluding studies where data were estimated; (3) Analyzing only RCTs and excluding single-group pre-post studies; (4) Changing the meta-analysis statistical model (fixed-effect model *vs*. random-effects model).

### 2.10 Assessment of publication bias

According to methodological recommendations, funnel plots can be effectively utilized for publication bias assessment when the number of studies included in a meta-analysis is typically ≥10. In this study, neither the overall analysis nor the primary subgroup analyses met this threshold, and therefore funnel plots or related quantitative tests (e.g., Egger’s test) were not performed. In the discussion section, we will conduct a qualitative discussion on potential publication bias by integrating the literature screening process (e.g., the presence of a substantial amount of unpublished gray literature) with the characteristics of the study results (e.g., small sample size effects).

## 3 Results

### 3.1 Characteristics of included studies

**Table 2** presents the basic characteristics of the 12 included studies (comprising a total of 520 children aged 3–12 years) conducted between 2010 and 2025 across multiple countries or regions. All studies utilized standardized diagnostic tools. The specific intervention types, dosages, and designs are detailed in **Table 2**.

**Table 2:** Basic characteristics of included studies.

Study	Country	Diagnostic	Intervention	Study design	E/C	Total minute	Week	Frequency	Single duration	Outcome indicator
(21)	China	ABC	Android intervention combined with motion-sensing games	RCT	32/32	720	12	1 time/week	60 min	CARS
(22)	China	DSM-V, ADOS-2	physical play-based Games	RCT	15/15	1440	8	6 times/week	30min	EF: WM\IC\CF
(23)	China	DSM-5, CARS	Structured Martial Arts Games	RCT	28/28	4320	24	3 times/week	60 min	1.ATEC 2.ABC 3.GMFM
(24)	China	ABC	physical play-based Games	QE	30/10	2160	12	3 times/week	60 min	ABC
(25)	China	DSM-5	Orff Music and physical play-based games	RCT	12/10	1620	9	3 times/week	60 min	SRS
(26)	China	DSM-5	Group physical play-based Games	RCT	36/36	3600	12	5 times/week	60 min	SRS
(27)	Iran	ADOS-2	Kinect	RCT	20/20	840	8	3 times/week	35min	1.MABC-2 2.WCST
(28)	China Taiwan	DSM-IV	WESP	COT	8/8	1800	10	2 times/week	90min	1.HAAR 2.SSBS-2
(29)	China Taiwan	DSM-IV	Table tennis training intervention	COT	11/11	1480	12	2 times/week	70min	WCST
(30)	China	CARS	1.Ball Combination Training Program (BCTP) 2.Mini-basketball Training Program (MBTP)	RCT	14/14	2770	12	5 times/week	45min	SRS-2
(31)	China	DSM-IV	Kinect	RCT	35/35	960	8	4 times/week	30min	PEDI
(32)	China	ASD	Motion Sensing Games	RCT	30/30	240	8	1 time/week	30min	PEDI

ABC, Autism Behavior Checklist, a screening tool used to identify children who may have autism by assessing their behaviors. ABC, Aberrant Behavior Checklist, a scale for quantifying problem behaviors (e.g., hyperactivity, aggression) in individuals with developmental disabilities to aid treatment monitoring. ADOS-2, Autism Diagnostic Observation Schedule, Second Edition, the “gold standard” instrument for diagnosing autism spectrum disorder (ASD) through direct observation. TEC, Autism Treatment Evaluation Checklist, a questionnaire measuring intervention effectiveness for children with autism by quantifying progress in language, social skills, and behavior. ASD, Autism Spectrum Disorder, a developmental disorder characterized by difficulties in social interaction and communication, along with restricted and repetitive patterns of behavior. CARS, Childhood Autism Rating Scale, a standardized tool assessing the presence and severity of autism spectrum disorder (ASD) in children by evaluating behaviors related to social interaction, communication, and restricted interests. DSM-IV, Diagnostic and Statistical Manual of Mental Disorders, Fourth Edition, the classification standard for mental disorders used by mental health professionals before 2013. DSM-V, Diagnostic and Statistical Manual of Mental Disorders, Fifth Edition, the current standard classification manual for mental disorders, providing diagnostic criteria for mental health professionals. EF, WM\IC\CF, Executive Function (EF) components including Working Memory (WM), Inhibitory Control (IC), and Cognitive Flexibility (CF); core cognitive processes regulated by the prefrontal cortex, critical for goal-directed behavior and commonly assessed in neurodevelopmental disorders. GMFM, Gross Motor Function Measure, a tool evaluating gross motor skills (e.g., sitting, walking) in children with cerebral palsy or neuromuscular conditions to track rehabilitation progress. HAAR, High-functioning Autism Assessment Rating Scale, a scale presumably designed to evaluate social and adaptive skills in individuals with high-functioning autism spectrum disorder. MABC-2, Movement Assessment Battery for Children, 2nd Edition, a standardized test identifying motor coordination difficulties in children aged 4–16, used to diagnose developmental coordination disorder. PEDI, Pediatric Evaluation of Disability Inventory, a measure of functional independence in daily activities (self-care, mobility, social participation) for children with disabilities, guiding rehabilitation planning. SRS, Social Responsiveness Scale, a rating scale assessing social impairment in individuals with autism spectrum disorder, measuring difficulties in social interaction and communication. SRS-2, Social Responsiveness Scale, 2nd Edition, an updated version of the SRS with expanded norms, assessing social impairment across the autism spectrum in children and adults. WCST, Wisconsin Card Sorting Test, a neuropsychological task evaluating cognitive flexibility and abstract reasoning, often used to assess frontal lobe function in individuals with brain injury or psychiatric conditions.

The 12 included studies involved a total of 520 children aged 3–12 years. Regarding gender, most studies reported a higher proportion of males, which is consistent with the epidemiology of ASD; however, exact numbers were not consistently reported across all studies. Diagnostic confirmation was based on standardized tools such as ADOS-2, ADI-R, DSM-5 criteria, or CARS. Information on ASD severity and intellectual functioning was provided in only a few studies: for example, Fu and Shi (23) included children with mild to moderate symptoms, and Milajerdi et al. (27) explicitly excluded participants with severe cognitive impairment. The lack of uniform reporting of these characteristics across studies precluded subgroup analyses based on severity or cognitive level.

### 3.2 Risk of bias assessment

**Figure 2:**
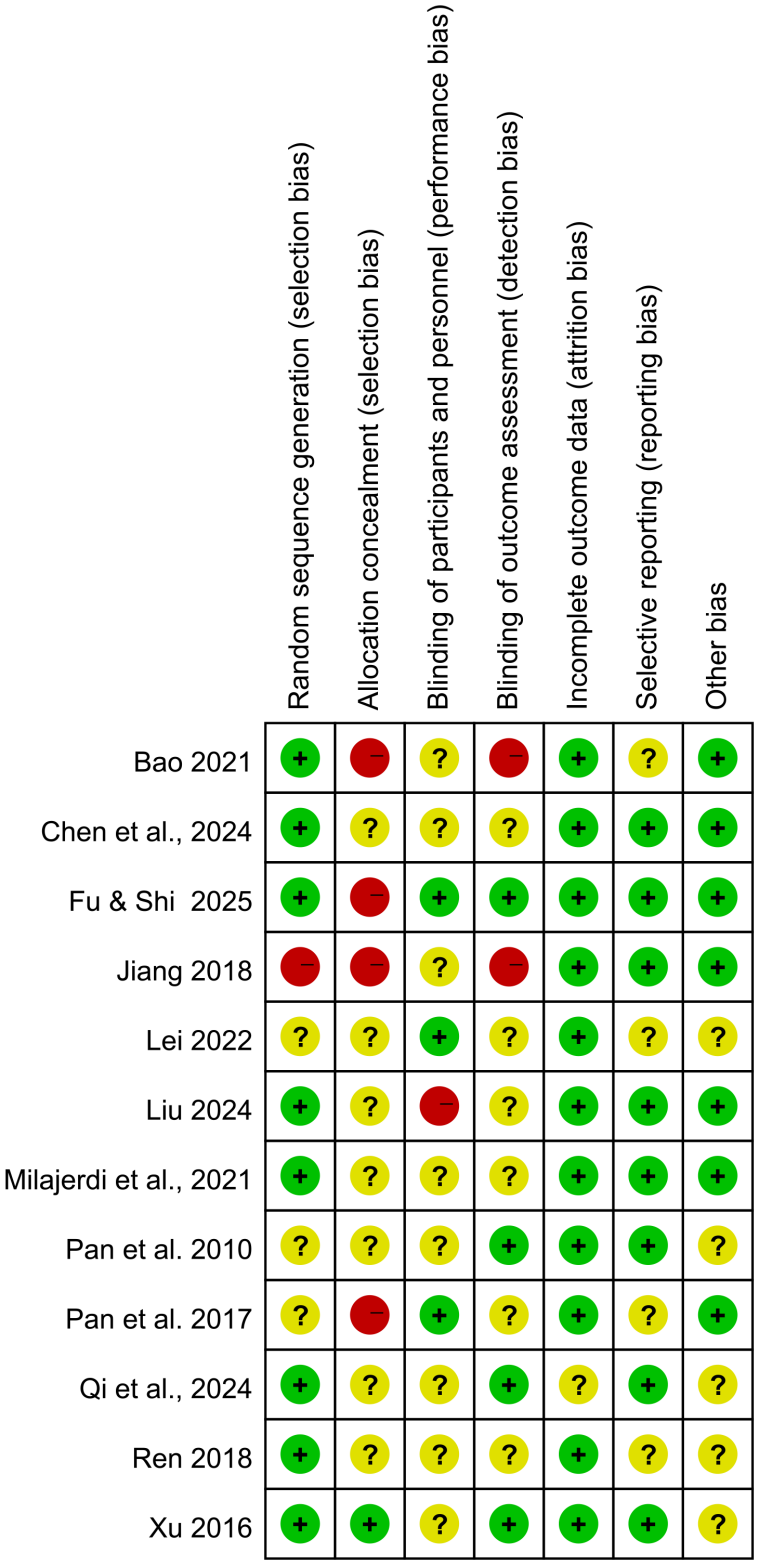
Risk of bias graph.

**Figure 3:**
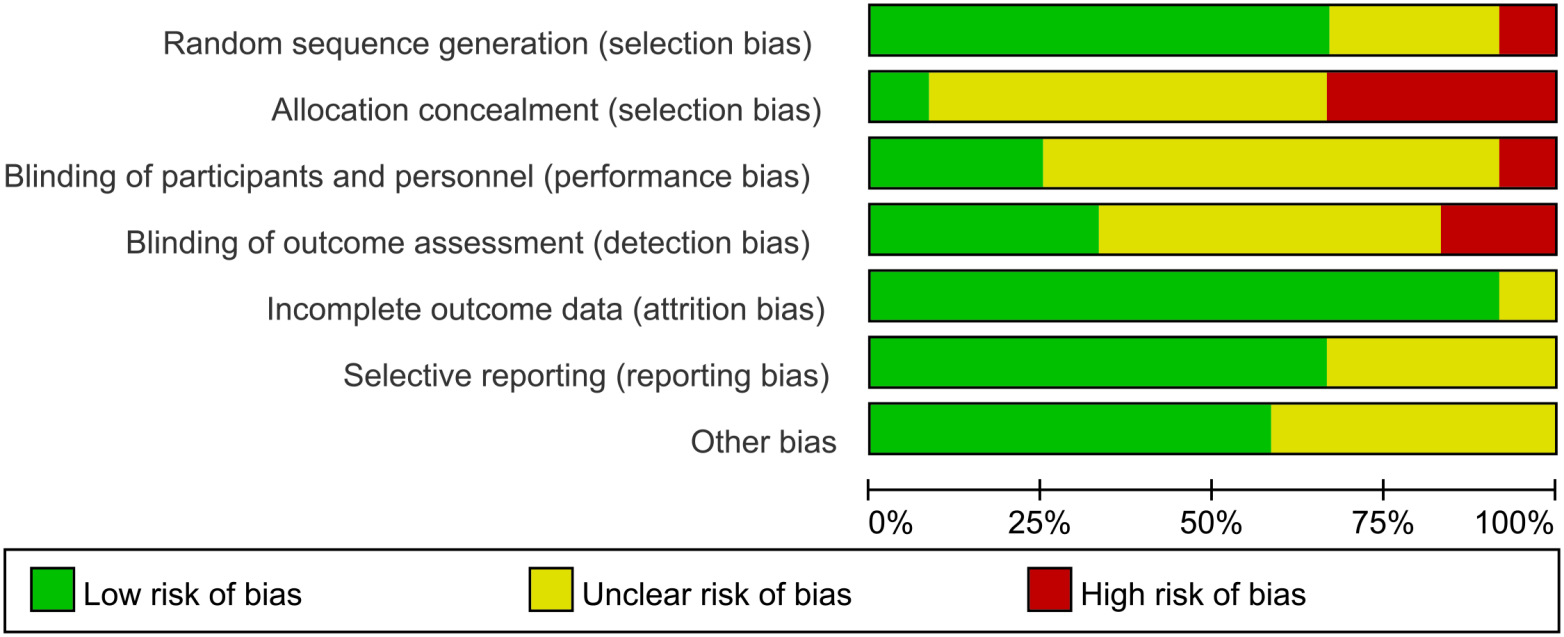
Summary of risk of bias.

**Figures 2** and **3** illustrate the risk of bias across the included studies. Among the seven domains assessed, concerns were identified in some of the 12 studies, primarily regarding allocation concealment (selection bias) and blinding of participants and personnel (performance bias). The included studies demonstrated a low risk of bias in the domains of random sequence generation, selective reporting, and, for some studies, blinding of outcome assessment.

### 3.3 Meta-analysis results and sensitivity analysis

This study employed a random-effects model to comprehensively evaluate the effects of physical play-based games on the executive functions (EFs) and social behavior of children with ASD. The specific results are as follows:

#### 3.3.1 Executive function outcomes

**Figure 4:**
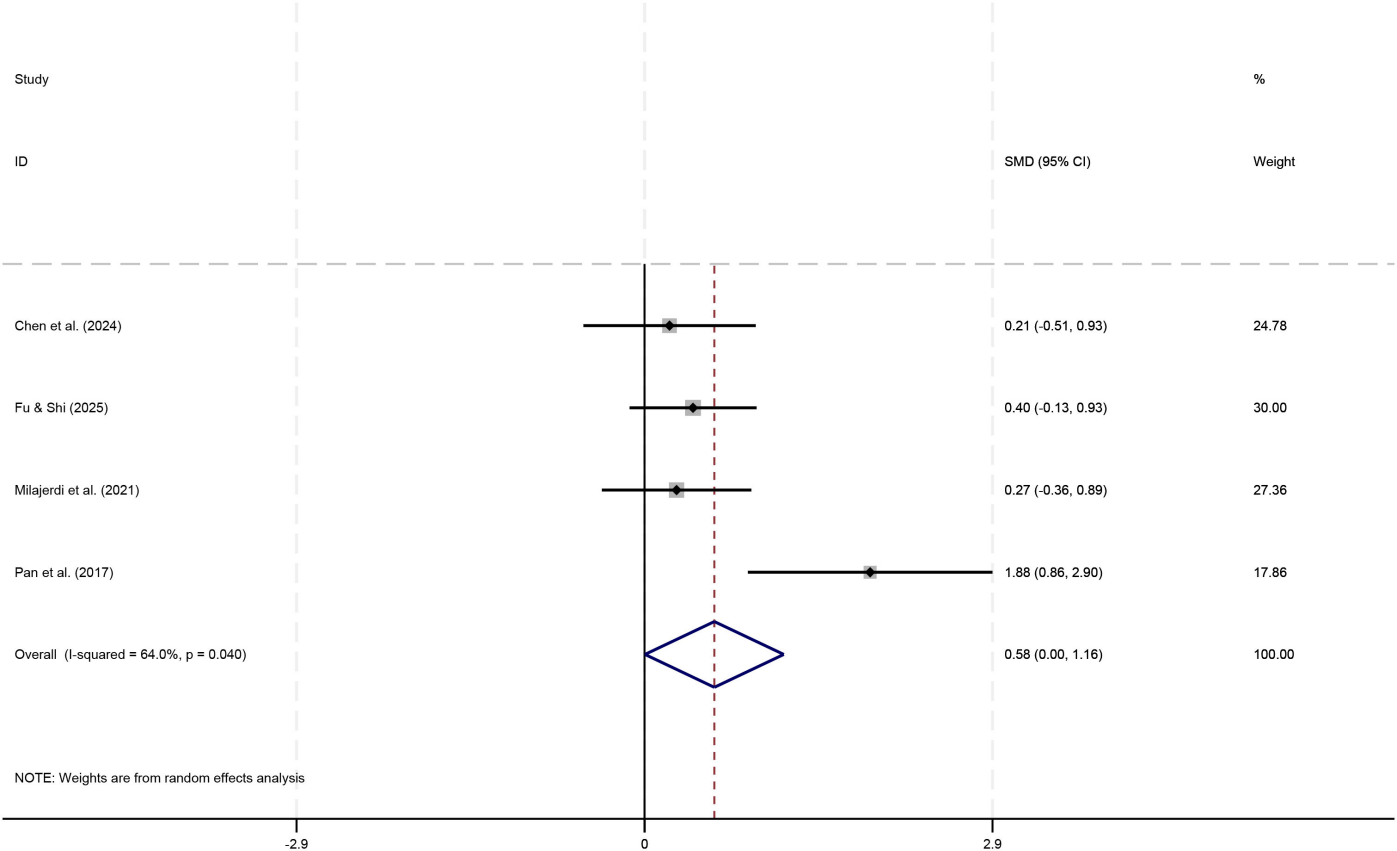
Forest plot for executive functions.

In the executive function section, a total of 4 studies (n=148) were retrieved investigating the effects of physical play-based Games interventions on executive function in children with ASD. The preliminary Meta-analysis results in **Figure 4** initially showed a combined effect size of SMD = 0.58 (95% CI: 0.00,1.16), with the lower limit of the 95% confidence interval being 0 (P≈0.05). Although the point estimate (SMD = 0.58) indicated an upward trend in post-intervention executive function scores, its 95% confidence interval included 0, and the P-value was at the significance boundary (P≈0.05), suggesting that the improvement trend was not statistically significant. This result was influenced by significant heterogeneity (I²=64.0%, P = 0.040), indicating substantial differences in effect sizes across studies. Further examination revealed that the study by Pan et al. (29) exhibited an abnormally high effect size (SMD = 1.88), which may be the primary source of heterogeneity.

**Figure 5:**
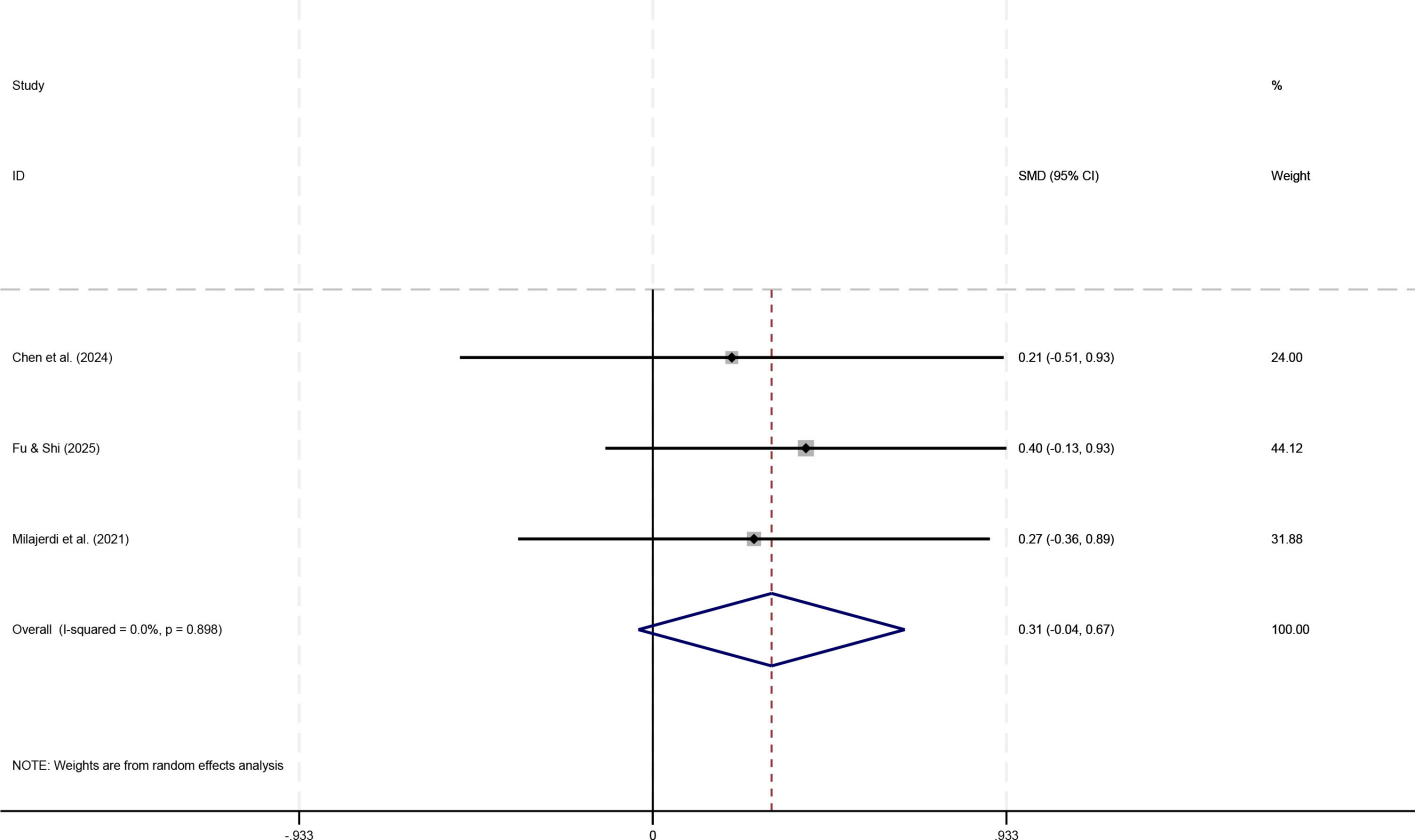
Forest plot for EFs after removing the outlier study.

To test the consistency of the findings, a sensitivity analysis was conducted by removing this study and re-pooling the data. The results (**Figure 5**) showed that the meta-analysis effect size for the remaining 3 studies decreased to SMD = 0.31 (95% CI: -0.04, 0.67). The confidence interval now included 0, rendering the result statistically non-significant (P > 0.05). Furthermore, heterogeneity between studies was completely eliminated (I² = 0.0%, P = 0.688), indicating highly consistent findings among the remaining studies.

In summary, current evidence suggests that physical play-based game interventions may have only a small, statistically non-significant improving trend on the executive functions of children with ASD (SMD ≈ 0.3). Although the results of the remaining studies are consistent, the limited sample size results in insufficient statistical power to draw a definitive conclusion regarding effectiveness. Therefore, more high-quality, large-scale studies are needed in the future for further verification.

#### 3.3.2 Social behavior outcomes

**Figure 6:**
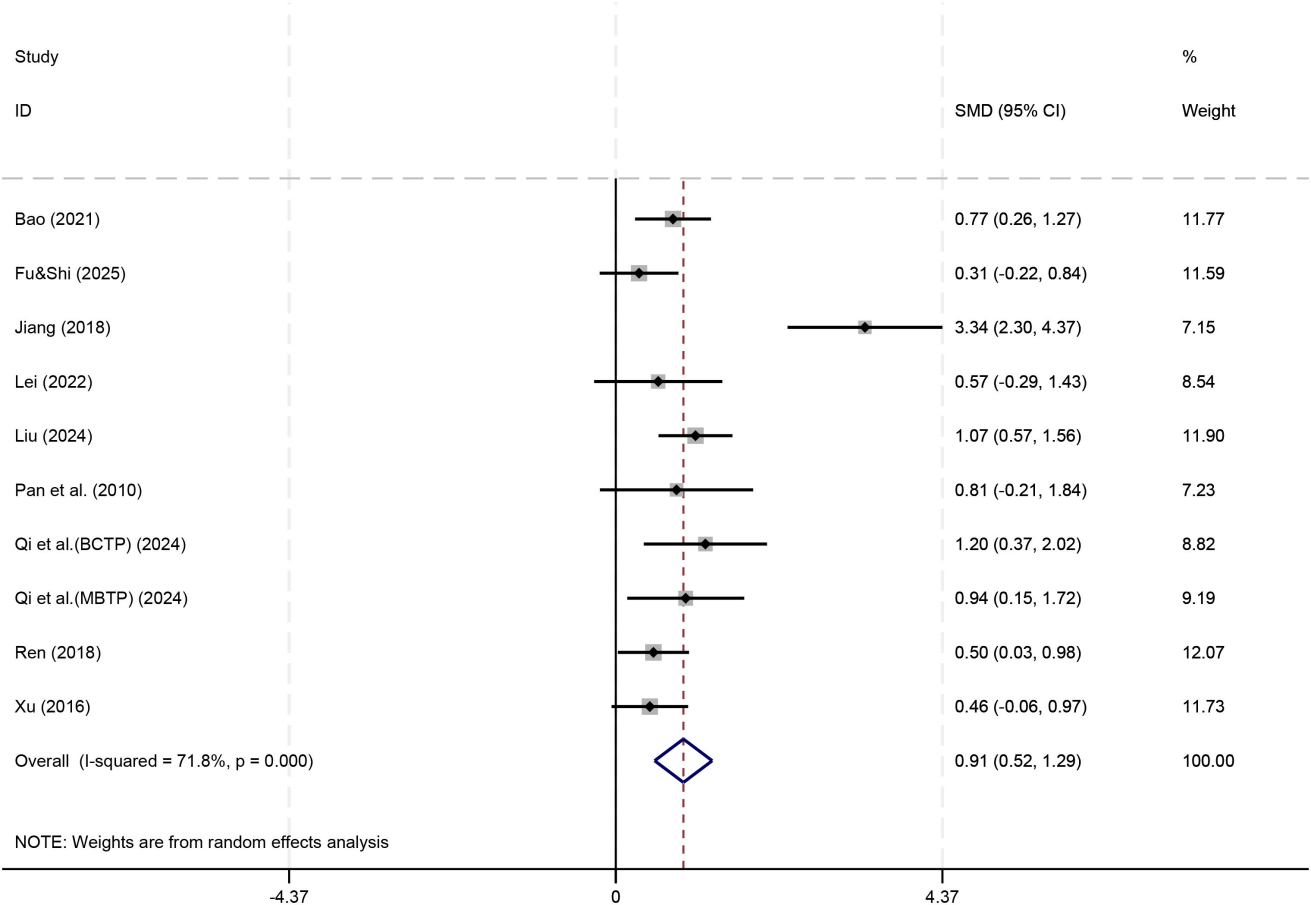
Forest plot for social behavior.

In the social behavior section, a total of 10 studies (n=455) were included to investigate the effects of physical play-based Games interventions on social behavior in children with ASD. The meta-analysis results in **Figure 6** showed that the pooled effect size was statistically significant (SMD = 0.91,95% CI [0.52,1.29], P <0.001). However, high heterogeneity was observed among the studies (I² = 71.8%, P <0.001), indicating significant variation in effect sizes across different studies. This heterogeneity may stem from differences in intervention protocols (e.g., game types), intervention dosage (frequency, duration), or the social behavior assessment tools used. Further examination revealed that the study by Jiang et al. (24) exhibited an abnormally prominent effect size (SMD = 3.34), which may be the primary source of heterogeneity.

**Figure 7:**
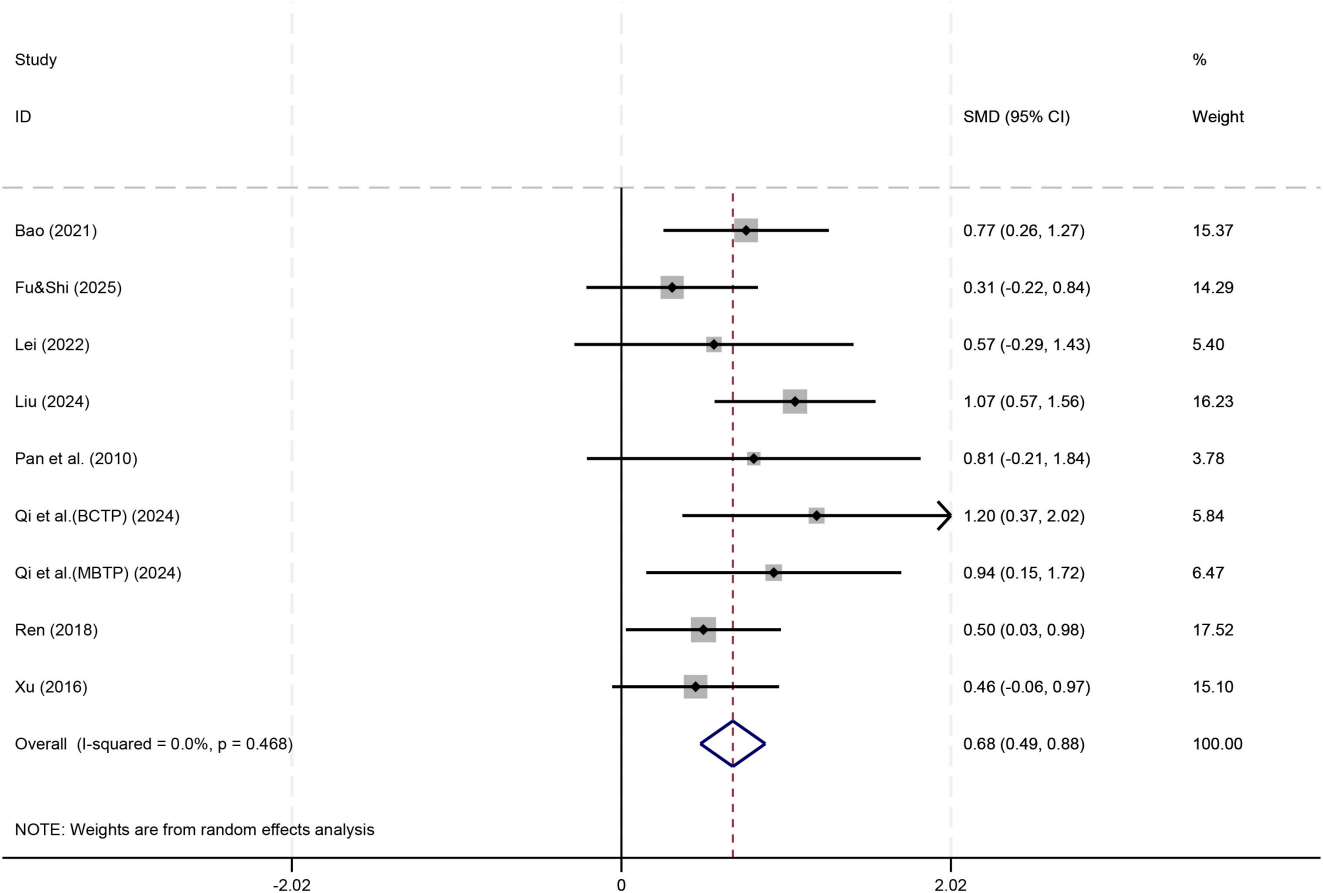
Forest plot for social behavior after removing the outlier study.

To obtain a more consistent conclusion, a sensitivity analysis was performed by excluding this study and re-pooling the results of the remaining 9 studies, as shown in **Figure 7**. The results revealed an adjusted effect size of SMD = 0.68 (95% CI [0.49, 0.88]), which remained statistically significant (P < 0.001). Furthermore, heterogeneity between studies was completely eliminated (I² = 0.0%, P = 0.468), indicating highly consistent findings among the remaining studies.

In summary, the most consistent current evidence suggests that physical play-based game interventions can significantly improve the social behavior of children with ASD, with a medium effect size (SMD = 0.68). This conclusion is supported by a high degree of consistency across different studies.

### 3.4 Subgroup analysis

This study conducted subgroup analyses to further explore potential moderating factors influencing the effectiveness of physical play-based games in improving social behavior in children with ASD. Detailed results are presented in **Table 3**.

**Table 3:** Subgroup analysis of social behavior by intervention type, frequency, and session duration of physical play-based games.

Adjusting variable	Subgroups category	Study	SMD (95% CI)	I², P	P for subgroup difference
Type	Concerted	3	0.61 (0.27, 0.94)	I² = 0.0%, P = 0.698	0.21
	Traditional	7	1.08 (0.52, 1.65)	I² = 79.7%, P = 0.000	
Frequency	≥ 4times/week	7	1.09 (0.54, 1.63)	I² = 78.4%, P = 0.000	0.562
	< 4times/week	3	0.52 (0.19, 0.85)	I² = 0.0%, P = 0.828	
Single Duration	≥ 60min	6	0.78 (0.53, 1.03)	I² = 12.4%, P = 0.336	0.216
	< 60min	3	0.52 (0.19, 0.85)	I² = 0.0%, P = 0.828	

When intervention type was considered as a moderator, the results showed that Traditional Physical Play-based Game Interventions (7 studies) demonstrated a larger effect size (SMD = 1.08) for improving social behavior compared to Combined Interventions (3 studies, SMD = 0.61). However, the difference between the two groups did not reach statistical significance (P = 0.21). Furthermore, results within the Combined Intervention subgroup showed very high consistency (I² = 0.0%), whereas substantial heterogeneity remained within the Traditional Physical Play-based Game Intervention subgroup (I² = 79.7%). This suggests that the ‘traditional games’ category might encompass specific programs with varying degrees of effectiveness.

When weekly intervention frequency was used as a moderator, high-frequency interventions (≥4 sessions/week, 7 studies) yielded a larger effect size (SMD = 1.09) than low-frequency interventions (<4 sessions/week, 3 studies, SMD = 0.52), but again, the between-group difference was not statistically significant (P = 0.562). Results within the low-frequency subgroup were extremely homogeneous (I² = 0.0%), while high heterogeneity persisted within the high-frequency subgroup (I² = 78.4%).

When session duration was used as a moderator, longer-duration sessions (≥60 minutes, 6 studies) produced a larger effect size (SMD = 0.78) compared to shorter-duration sessions (<60 minutes, 3 studies, SMD = 0.52), but the difference was not statistically significant (P = 0.216). Heterogeneity within both duration subgroups was low (I² = 12.4% and I² = 0.0%, respectively), indicating relatively stable intervention effects across different session lengths.

It is important to note that the subgroup analyses in this study are exploratory and observational in nature. Although the data show a trend where intervention schemes characterized as ‘traditional,’ ‘high-frequency,’ and ‘long-duration’ are associated with higher estimated effect sizes, the lack of statistically significant differences between subgroups means we cannot definitively conclude that these moderator variables are decisive factors causing the effect variations. Furthermore, the statistical power of some comparisons may be limited due to the small number of studies in certain subgroups (e.g., Combined Interventions, low frequency, short duration). Therefore, the interpretation of these findings should be approached with caution.

Consequently, while these trends provide preliminary hypotheses for future research, they should not be used as a basis for prescriptive clinical recommendations regarding intervention dosage or type.

## 4 Discussion

### 4.1 Main findings

To our knowledge, this study is one of the earliest systematic meta-analyses comparing the effects of physical activity games on executive function and social behavior in children with ASD. The meta-analysis, which synthesized data from 12 studies involving 520 participants, demonstrated that physical play-based Games interventions had certain positive effects on executive function and social behavior in ASD children. The main findings are as follows:

In terms of executive function, current evidence indicates that physical play-based games interventions showed a small and statistically non-significant improvement effect (SMD ≈ 0.31, 95% CI [-0.04, 0.67]). This null finding may be attributable to the limited number of studies (n=4), small sample sizes, and heterogeneity in measurement tools; however, it may also indicate that the intervention has limited efficacy in this domain. Further research with larger samples and standardized outcome measures is needed to clarify this issue. Regarding social behavior, the meta-analysis results showed that physical activity game interventions significantly improved social behavior in ASD children (SMD = 0.68), indicating a moderate effect size. This result remained stable after sensitivity analysis and was consistent across different studies. Our finding that physical play-based games significantly improve social behavior (SMD = 0.68) aligns with the results of Li et al. (15), who also reported a moderate effect on social outcomes (15). In contrast, the non−significant trend for executive function in our analysis (SMD ≈ 0.31) is consistent with the inconclusive evidence summarized by Li et al., underscoring the need for further high−quality trials targeting executive functions.

Subgroup analysis results indicated that intervention models characterized as traditional physical play-based games, high frequency (≥4 sessions/week), and long single-session duration (≥60 minutes) numerically presented larger effect sizes. However, it is important to note that the between-group differences did not reach statistical significance, which may be associated with the small sample sizes within subgroups or heterogeneity among studies. The aforementioned subgroup analyses are all based on cross-study comparisons; the strength of evidence from such indirect comparisons is lower than that from direct head-to-head randomized controlled trials. Therefore, conclusions regarding effect differences among different exercise types and dosages from this meta-analysis should be interpreted with caution, and direct validation through head-to-head randomized controlled trials is still needed in the future.

### 4.2 Discussion of mechanisms

#### 4.2.1 Mechanisms for improving executive functions

The proposed mechanisms by which physical play-based games may enhance executive functions (EFs) in children with ASD center on activity-induced neural plasticity. A common pathway across interventions is the activation and strengthening of frontal lobe-mediated cognitive control networks through challenging physical activity (S. 33). Different game formats may engage these networks through distinct pathways: structured group activities (e.g., martial arts games) integrate EF demands within rule-based social-motor contexts, requiring inhibitory control, working memory, and cognitive flexibility (23). In contrast, exergames (e.g., Kinect-based activities) provide intensive ‘perception-decision-action’ feedback loops that specifically target response inhibition and working memory updating (27). These observations align with embodied cognition theories, which posit that cognitive processes are deeply rooted in bodily interactions with the environment. However, it is important to note that these mechanistic pathways are inferred from study designs and theoretical frameworks; none of the included studies directly tested these mechanisms (e.g., through neuroimaging or mediation analyses).

#### 4.2.2 Mechanisms for improving social behavior

The positive effects of physical play-based games on social behavior in children with ASD are likely mediated by several interacting pathways. First, structured game contexts (e.g., cooperative tasks, turn-taking games) provide predictable, low-anxiety environments that naturally elicit and reinforce social behaviors such as joint attention, communication, and cooperation (26, 28). Second, the embodied nature of physical activity may enhance social cognition through multisensory integration and improved self-regulation. For instance, aquatic activities provide tactile stimulation that may have calming effects (28), while coordinated movements in martial arts may strengthen neural circuits involved in both motor control and social perception (23). Third, group-based formats facilitate peer modeling and immediate social feedback, which are critical for skill generalization (26). Fourth, the enjoyable, gamified nature of these interventions increases intrinsic motivation and positive affect, potentially reducing social anxiety and increasing the frequency of social initiation attempts(S. 33). Crucially, these proposed mechanisms are theoretical; the included studies did not empirically test mediation pathways, and future research should directly examine how these interventions produce their effects.

#### 4.2.3 Dose-response relationship

Subgroup analysis in this study revealed that high frequency (≥4 times/week), prolonged duration (≥60 minutes per session), and traditional games demonstrated a trend toward greater effect sizes numerically. However, these differences among subgroups were not statistically significant. Therefore, these trends should be regarded as exploratory findings rather than definitive dose-response conclusions, providing preliminary clues for future research but insufficient as strong evidence for formulating specific intervention strategies. This trend aligns with the “repeated exposure and consolidation learning” theory: ASD children require more repetition and practice opportunities in the acquisition of social skills, and interventions with higher frequency and adequate single-session duration may facilitate skill automation and generalization (34). Additionally, traditional physical play-based Games exhibited larger effect sizes, likely due to their clear rules and structured nature, which better meet ASD children’s need for predictable environments, thereby reducing anxiety and enhancing engagement.

### 4.3 Limitations

Although this study strived for rigor, several limitations remain that necessitate cautious interpretation of the results. Firstly, the number and quality of included studies constrain the certainty of the conclusions. Despite a comprehensive literature search, there is still a shortage of high-quality randomized controlled trials meeting the criteria, particularly concerning studies on EFs (only 4 studies). Some studies presented a risk of bias regarding allocation concealment and blinding of participants, which may have led to an overestimation of the intervention effect. Furthermore, the generally small sample sizes in the included studies may have resulted in insufficient statistical power, affecting the detection of small-to-medium effects, especially for effects on EFs.

Secondly, there was considerable clinical and methodological heterogeneity among the studies. Although heterogeneity for the social behavior outcome decreased to an acceptable level after removing outliers, the sources of heterogeneity still warrant attention. There were significant variations across studies in intervention protocols (e.g., martial arts, aquatic activities, exergames), dosage (frequency, duration, total period), implementation setting (individual/group), and control group design (blank control, usual activity). Although we conducted subgroup analyses to explore some moderating factors, conclusive dose-response conclusions could not be drawn due to the limited number of studies within subgroups. Moreover, different studies employed diverse assessment tools to measure executive functions and social behavior. These tools vary in sensitivity, focus, and scoring direction, which may pose challenges for result synthesis. This heterogeneity has important implications for the interpretation of our findings. First, the diversity of intervention protocols means that our pooled effect size represents an average across qualitatively different types of ‘physical play-based games,’ potentially masking which specific types are most effective. Second, the use of different assessment tools (e.g., SRS, ABC, CARS) across studies introduces measurement-related variability, as these tools may tap into different aspects of social behavior and have varying sensitivity to change. Third, studies conducted in different cultural contexts (e.g., Mainland China, Taiwan, Iran) may reflect cultural differences in how social behaviors are expressed, perceived, and evaluated. These factors collectively limit the precision of our effect estimates and the generalizability of our conclusions. Future research should aim to standardize intervention protocols and outcome measures, and explicitly examine cultural and contextual moderators.

Fourth, the exploration of potential moderating variables was not exhaustive. This study primarily focused on the characteristics of the intervention itself. However, significant individual differences among children with ASD, such as age, symptom severity, intellectual level, and baseline motor ability, may substantially moderate the intervention effects. Limited by the information reported in the original studies, this research could not conduct in-depth subgroup analyses or meta-regression on these important individual characteristics.

Finally, the long-term effects and generalization effects remain unclear. Follow-up assessments in the vast majority of included studies were conducted immediately post-intervention, lacking medium- to long-term follow-up data (e.g., 3 months, 6 months, or longer). Therefore, whether the improvements brought about by physical play-based games can be maintained after the intervention ends and whether they can generalize to natural settings such as daily life and school still require future research for confirmation.

### 4.4 Implications and future directions

Through systematic review and meta-analysis, this study provides the first comprehensive evaluation of the intervention effects of physical play-based games on the executive functions and social behavior of children with ASD. The results indicate that physical play-based games are a non-pharmacological intervention with a medium effect size for improving the social behavior of children with ASD, offering preliminary evidence for their application in rehabilitation practice. Although the improvement effect on executive functions did not reach significance, a positive trend was observed, pointing the way for subsequent research. This study also preliminarily explored the possible influence of dosage factors such as intervention type, frequency, and duration, providing a reference for the refined design of future intervention programs.

Based on the findings and limitations of this study, future research can delve deeper in the following aspects: Firstly, actively conduct high-quality, large-sample randomized controlled trials, prioritizing rigorous methodological design and expanding sample sizes to enhance statistical power and the consistency of conclusions. Secondly, promote the standardization of intervention protocols and exploration of mechanisms, while combining neurophysiological techniques and behavioral measures to investigate the cognitive-neural mechanisms and behavioral pathways underlying the effects of physical play-based games on children with ASD. Thirdly, conduct in-depth investigations into the dose-response relationship and individual differences. Future research should systematically manipulate and report intervention dosages and adopt a dose-response analysis framework, while paying attention to the heterogeneity among children with ASD. Finally, assess long-term effects and ecological validity by incorporating medium- to long-term follow-up assessments and employing more diversified assessment methods to examine the maintenance of intervention effects and their generalization to real-life scenarios.

## 5 Conclusion

This study demonstrates that physical activity-based games may serve as a potential intervention to improve social behavior in children with ASD, with moderate effect sizes. However, given the limited number of studies included and methodological heterogeneity, these findings should be interpreted with caution. Future high-quality research is needed to validate its efficacy, identify optimal intervention approaches, and explore its potential impacts on related domains such as executive function. Regarding executive function, the current evidence, based on a limited number of studies, shows only a small and statistically non-significant improving trend (SMD ≈ 0.31). Therefore, no definitive conclusions can be drawn about the efficacy of physical play-based games on executive functions in children with ASD at this stage. Among them, traditional physical play-based games, characterized by being rule-based, physically interactive, and group-oriented, demonstrated the most positive improvement effects. They effectively promote the comprehensive abilities of children with ASD in areas such as social responsiveness, initiation of interactions, and cooperative behaviors.

Regarding intervention dosage, exploratory subgroup analyses suggested a trend toward greater potential benefit for intervention models featuring higher frequency (≥4 sessions per week) and longer session duration (≥60 minutes per session). However, as these findings are based on underpowered subgroup comparisons and were not statistically significant, they should be viewed as preliminary observations requiring confirmation, rather than definitive dosage recommendations. While observed differences in effects based on intervention type and dosage present as trends, they suggest that in practice, priority should be given to evidence-supported and well-structured physical activity programs. Furthermore, these programs require personalized design and dynamic adjustment based on the child’s individual interests, functional baseline, and specific social goals.

This study provides important empirical support for the systematic integration of physical play-based games as a structured, evidence-based, and easily scalable non-pharmacological intervention strategy into the rehabilitation and educational support systems for children with ASD.

## Data Availability

The original contributions presented in the study are included in the article/**Supplementary Material**. Further inquiries can be directed to the corresponding author.

## References

[1] Baio J . Prevalence of autism spectrum disorder among children aged 8 years — Autism and Developmental Disabilities Monitoring Network, 11 sites, United States 2014. MMWR Surveill Summaries. (2018) 67:1–23. doi: 10.15585/mmwr.ss6706a1. PMID: 29701730 PMC5919599

[2] Lord C Brugha TS Charman T Cusack J Dumas G Frazier T . Autism spectrum disorder. Nat Rev Dis Primers. (2020) 6:1–23. doi: 10.1038/s41572-019-0138-4. PMID: 31949163 PMC8900942

[3] Lord C Brugha TS Charman T Cusack J Dumas G Frazier T . Autism spectrum disorder. Nat Rev Dis Primers. (2020) 6:5. doi: 10.1038/s41572-019-0138-4. PMID: 31949163 PMC8900942

[4] Gong L Wang T . Research progress on social maintenance defects of individuals with autism spectrum disorder. Chinese Journal of Special Education. Beijing: Chinese National Academy of Educational Sciences (2022). p. 3:1–8. Available online at: https://zgtsjy.cnaes.edu.cn/ (Accessed March 10, 2026).

[5] Donnelly JE Hillman CH Castelli D Etnier JL Lee S Tomporowski P . Physical activity, fitness, cognitive function, and academic achievement in children: a systematic review. Med Sci Sports Exerc. (2016) 48:1197–222. doi: 10.1249/MSS.0000000000000901. PMID: 27182986 PMC4874515

[6] Diamond A . Executive functions. Annu Rev Psychol. (2013) 64:135–68. doi: 10.1146/annurev-psych-113011-143750. PMID: 23020641 PMC4084861

[7] Cristofori I Cohen-Zimerman S Grafman J . “ Executive functions”. In: D'Esposito M Grafman JH , editors. The Frontal Lobes. 3rd ed. New York: Elsevier (2019) p. 197–220. doi: 10.1016/B978-0-12-804281-6.00011-2, PMID: 31590731

[8] Lord C MaGill-Evans J . Peer interactions of autistic children and adolescents. Dev Psychopathol. (1995) 7:611–26. doi: 10.1017/S095457940000674X. PMID: 41694064

[9] Geannopoulos ZF Moody CT McGregor HA Baertschi D Bates S Laugeson EA . Outcomes in PEERS® for adolescents across neurodevelopmental disorders: ADHD, autism, and their co-occurrence. Adv Neurodev Disord. (2024) 8:614–26. doi: 10.1007/s41252-023-00380-z. PMID: 41784122

[10] Shahane V Kilyk A Srinivasan SM . Effects of physical activity and exercise-based interventions in young adults with autism spectrum disorder: A systematic review. Autism. (2024) 28:276–300. doi: 10.1177/13623613231169058. PMID: 37128159

[11] Mundy P Bullen J . The bidirectional social-cognitive mechanisms of the social-attention symptoms of autism. Front Psychiatry. (2022) 12:752274. doi: 10.3389/fpsyt.2021.752274. PMID: 35173636 PMC8841840

[12] Tengda . Exploration on basic theories of sports games. J Beijing Sport Univ. (2005), 260–2. doi: 10.19582/j.cnki.11-3785/g8.2005.02.043

[13] Howells K Sivaratnam C May T Lindor E McGillivray J Rinehart N . Efficacy of group-based organised physical activity participation for social outcomes in children with autism spectrum disorder: A systematic review and meta-analysis. J Autism Dev Disord. (2019) 49:3290–308. doi: 10.1007/s10803-019-04050-9. PMID: 31102193

[14] Zhao M Chen S . The effects of structured physical activity program on social interaction and communication for children with autism. BioMed Res Int. (2018) 2018:1825046. doi: 10.1155/2018/1825046. PMID: 29568743 PMC5820623

[15] Li Z Ramli S Romli M Ahmad Azmeer R Sulaiman PS Lin C . Focus group discussion on factors for development of somatosensory games on hand function training for self-care ability of children with autism. Int J Dev Disabil. (2024) 71(7):1048–60. doi: 10.1080/20473869.2023.2301188. PMID: 41262118 PMC12624937

[16] Higgins JP Altman DG Gøtzsche PC Jüni P Moher D Oxman AD . The Cochrane Collaboration’s tool for assessing risk of bias in randomised trials. BMJ. (2011) 343:d5928. doi: 10.1136/bmj.d5928. PMID: 22008217 PMC3196245

[17] Salanti G Kavvoura FK Ioannidis JPA . Exploring the geometry of treatment networks. Ann Internal Med. (2008) 148:544–53. doi: 10.7326/0003-4819-148-7-200804010-00011. PMID: 18378949

[18] Swallow KM Jiang YV . Attentional load and attentional boost: A review of data and theory. Front Psychol. (2013) 4:274. doi: 10.3389/fpsyg.2013.00274. PMID: 23730294 PMC3657623

[19] van den Berk-Smeekens I de Korte MWP van Dongen-Boomsma M Oosterling IJ den Boer JC Barakova EI . Pivotal Response Treatment with and without robot-assistance for children with autism: A randomized controlled trial. Eur Child Adolesc Psychiatry. (2022) 31:1871–83. doi: 10.1007/s00787-021-01804-8. PMID: 34106357 PMC9663375

[20] Pieters LE Deenik J de Vet S Delespaul P van Harten PN . Combining actigraphy and experience sampling to assess physical activity and sleep in patients with psychosis: A feasibility study. Front Psychiatry. (2023) 14:1107812. doi: 10.3389/fpsyt.2023.1107812. PMID: 36911128 PMC9996223

[21] Bao . The application of humanoid robot intervention combined with body-sensing games in the rehabilitation treatment of children with autism spectrum disorder. Chin Convalescent Med. (2021) 30:157–8. doi: 10.13517/j.cnki.ccm.2021.02.015

[22] Chen H Liang Q Wang B Liu H Dong G Li K . Sports game intervention aids executive function enhancement in children with autism - an fNIRS study. Neurosci Lett. (2024) 822:137647. doi: 10.1016/j.neulet.2024.137647. PMID: 38242348

[23] Fu X Shi P . The intervention effect of structured martial arts games on behavioral impairments and motor functions in children with autism spectrum disorder. Front Psychol. (2025) 16:1660040. doi: 10.3389/fpsyg.2025.1660040. PMID: 41079876 PMC12510954

[24] Jiang F Xu D . Research on the intervention effect of physical activities on children with autism: A case study of Nanjing Ningxin Sunshine Home Disabled Persons’ Service Center. Sichuan Sports Sci. (2018) 37:51–4. doi: 10.13932/j.cnki.sctykx.2018.03.14

[25] Lei DX Liu Y Jiang M . An intervention study on the social skills of children with mild to moderate asd using Orff music combined with sports games. Bull Sports Sci Technol Literature. (2022) 30:87–90. doi: 10.19379/j.cnki.issn.1005-0256.2022.03.022

[26] Liu . The influence of group sports game intervention on the social skills and quality of life of children with autism spectrum disorder. School Hyg China. (2024) 45:110–4. doi: 10.16835/j.cnki.1000-9817.2024003

[27] Milajerdi HR Sheikh M Ghayour Najafabadi M Saghaei B Naghdi N Dewey D . The effects of physical activity and exergaming on motor skills and executive functions in children with autism spectrum disorder. Games Health J. (2021) 10:33–42. doi: 10.1089/g4h.2019.0180. PMID: 33370161

[28] Pan C-Y . Effects of water exercise swimming program on aquatic skills and social behaviors in children with autism spectrum disorders. Autism: Int J Res Pract. (2010) 14:9–28. doi: 10.1177/1362361309339496. PMID: 20124502

[29] Pan C-Y Chu C-H Tsai C-L Sung M-C Huang C-Y Ma W-Y . The impacts of physical activity intervention on physical and cognitive outcomes in children with autism spectrum disorder. Autism. (2017) 21:190–202. doi: 10.1177/1362361316633562. PMID: 27056845

[30] Qi K Liu Y Wang Z Xiong X Cai K Xu Y . Recreational ball games are effective in improving social communication impairments among preschoolers diagnosed with autism spectrum disorder: A multi-arm controlled study. BMC Sports Sci Med Rehabil. (2024) 16(1):176. doi: 10.1186/s13102-024-00957-8. PMID: 39175073 PMC11342502

[31] Ren . The influence of motion-sensing game rehabilitation therapy on social disorders, daily living abilities and emotional behaviors of children with autism. Chin J Health Psychol. (2018) 26:509–13. doi: 10.13342/j.cnki.cjhp.2018.04.008

[32] Xu . The utility of motion-sensing games in the intervention of children with autism. Chinese J Clin Psychol. (2016) 24:762–5. doi: 10.16128/j.cnki.1005-3611.2016.04.042

[33] Chen S Jing L Li C Wang H . Exploring the nexus between moderate-to-vigorous physical activity, self-disclosure, social anxiety, and adolescent social avoidance: Insights from a cross-sectional study in Central China. Children. (2023) 11:56–72. doi: 10.3390/children11010056. PMID: 38255369 PMC10814873

[34] Lang R Koegel LK Ashbaugh K Regester A Ence W Smith W . Physical exercise and individuals with autism spectrum disorders: A systematic review. Res Autism Spectr Disord. (2010) 4:565–76. doi: 10.1016/j.rasd.2010.01.006. PMID: 41783259

