# Supplementary material for: The impact of physical play-based games on executive functions and social behaviors in children with autism spectrum disorder: a systematic review and meta-analysis
Source: Front Psychiatry. 2026 Mar 23;17:1782760. doi: 10.3389/fpsyt.2026.1782760 (PMC13051336; doi:10.3389/fpsyt.2026.1782760)
Supplement: Supplementary file 2 [file Table1.docx]

Appendix: Full Search Strategies for Each Database

Search date: From inception to December 20, 2025

1. CNKI (China National Knowledge Infrastructure)

Search syntax: (SU='autism'+'autistic disorder'+'ASD') AND (SU='physical play'+'physical activity'+'exercise intervention'+'physical training'+'physical activity'+'sensory integration games'+'motion-sensing games') AND (SU='executive function'+'cognitive control'+'inhibitory control'+'working memory'+'cognitive flexibility'+'planning ability'+'attention') AND (SU='social behavior'+'social skills'+'social interaction'+'social communication'+'social responsiveness'+'peer interaction') AND (SU='children'+'preschool children'+'preschoolers')

1. Wanfang Database

Search syntax: (Subject: ("autism"+"autistic disorder"+"ASD")) AND (Subject: ("physical play"+"physical activity"+"exercise intervention"+"physical training"+"physical activity"+"sensory integration games"+"motion-sensing games")) AND (Subject: ("executive function"+"cognitive control"+"inhibitory control"+"working memory"+"cognitive flexibility"+"planning ability"+"attention")) AND (Subject: ("social behavior"+"social skills"+"social interaction"+"social communication"+"social responsiveness"+"peer interaction")) AND (Subject: ("children"+"preschool children"+"preschoolers"))

1. PubMed

("Autism Spectrum Disorder"[Mesh] OR "autis"[tiab] OR "ASD"[tiab] OR "Asperger"[tiab] OR "autistic disorder"[tiab]) AND ("Physical Play"[tiab] OR "physical activity"[tiab] OR "exercise"[Mesh] OR "exercise"[tiab] OR "sport"[tiab] OR "physical game"[tiab] OR "play-based intervention"[tiab] OR "recreational therapy"[tiab] OR "Kinect"[tiab] OR "exergam"[tiab] OR "motor intervention"[tiab]) AND ("Executive Function"[Mesh] OR "executive function"[tiab] OR "cognitive control"[tiab] OR "inhibitory control"[tiab] OR "working memory"[tiab] OR "cognitive flexibility"[tiab] OR "planning"[tiab] OR "attention control"[tiab]) AND ("Social Behavior"[Mesh] OR "social behavior"[tiab] OR "social skill"[tiab] OR "social interaction"[tiab] OR "social communication"[tiab] OR "social responsiveness"[tiab] OR "peer interaction"[tiab]) AND ("Child"[Mesh] OR "child"[tiab] OR "preschool"[tiab] OR "kid"[tiab] OR "toddler"[tiab])

1. Cochrane Library

#1 MeSH descriptor: [Autism Spectrum Disorder] explode all trees

#2 (autis* or ASD or asperger): ti,ab,kw

#3 #1 or #2

#4 MeSH descriptor: [Exercise] explode all trees

#5 ("physical play" or "physical activity" or exercise or sport or "physical game" or "play-based intervention" or "recreational therapy" or Kinect or exergam or "motor intervention"): ti,ab,kw

#6 #4 or #5

#7 MeSH descriptor: [Executive Function] explode all trees

#8 ("executive function" or "cognitive control" or "inhibitory control" or "working memory" or "cognitive flexibility" or planning or "attention control"): ti,ab,kw

#9 #7 or #8

#10 MeSH descriptor: [Social Behavior] explode all trees

#11 ("social behavior" or "social skill" or "social interaction" or "social communication" or "social responsiveness" or "peer interaction"): ti,ab,kw

#12 #10 or #11

#13 MeSH descriptor: [Child] explode all trees

#14 (child or preschool or kid or toddler*): ti,ab,kw

#15 #13 or #14

#16 #3 and #6 and #9 and #12 and #15

1. Web of Science

TS=(("Autism Spectrum Disorder" OR autis* OR ASD OR asperger)) AND TS=(("physical play" OR "physical activity" OR exercise OR sport OR "physical game" OR "play-based intervention" OR "recreational therapy" OR Kinect OR exergam OR "motor intervention")) AND TS=(("executive function" OR "cognitive control" OR "inhibitory control" OR "working memory" OR "cognitive flexibility" OR planning OR "attention control")) AND TS=(("social behavior" OR "social skill" OR "social interaction" OR "social communication" OR "social responsiveness" OR "peer interaction")) AND TS=((child OR preschool OR kid OR toddler*))

1. Embase

('autism'/exp OR 'autism spectrum disorder'/exp OR 'asperger syndrome'/exp OR 'autis':ti,ab OR 'asd':ti,ab) AND ('physical activity'/exp OR 'exercise'/exp OR 'kinesiotherapy'/exp OR 'physical play':ti,ab OR 'physical activity':ti,ab OR 'exercise':ti,ab OR 'sport':ti,ab OR 'physical game':ti,ab OR 'play-based intervention':ti,ab OR 'recreational therapy':ti,ab OR 'kinect':ti,ab OR 'exergam':ti,ab OR 'motor intervention':ti,ab) AND ('executive function'/exp OR 'executive function':ti,ab OR 'cognitive control':ti,ab OR 'inhibitory control':ti,ab OR 'working memory':ti,ab OR 'cognitive flexibility':ti,ab OR 'planning':ti,ab OR 'attention control':ti,ab) AND ('social behavior'/exp OR 'social interaction'/exp OR 'social communication'/exp OR 'social behavior':ti,ab OR 'social skill':ti,ab OR 'social interaction':ti,ab OR 'social communication':ti,ab OR 'social responsiveness':ti,ab OR 'peer interaction':ti,ab) AND ('child'/exp OR 'preschool child'/exp OR 'child':ti,ab OR 'preschool':ti,ab OR 'kid':ti,ab OR 'toddler*':ti,ab)

Table 1 The core concepts and keyword framework of the search strategy

| Core concept | English key words |
| --- | --- |
| Person | "Autism Spectrum Disorder"， autistic， ASD， asperger* |
| Intervention | “physical play-based Games”， “Physical Play”， Exercise， “Physical Activity”， Sport*， “Motor Intervention”， “Play-Based Intervention”， “Recreational Therapy” |
| Outcome1 | “Executive Function”， Cognitive Control， Inhibition， “Working Memory”， Cognitive Flexibility， Planning， Attention Control |
| Outcome 2 | “Social Behavior”， “Social Skills”， “Social Interaction”， “Social Communication”， “Social Responsiveness”， “Peer Interaction” |
| Range | child， preschool， kid， toddler* |
