# Supplementary material for: The impact of physical play-based games on executive functions and social behaviors in children with autism spectrum disorder: a systematic review and meta-analysis
Source: Front Psychiatry. 2026 Mar 23;17:1782760. doi: 10.3389/fpsyt.2026.1782760 (PMC13051336; doi:10.3389/fpsyt.2026.1782760)
Supplement: Supplementary file 3 [file Table2.docx]

**Identification of studies via databases and registers**

Records removed *before screening*:

Duplicate records removed (n =95 )

Records identified from*:

PubMed (n =293)

Cochrane Library (n =123)

Embase (n=38)

Web Of Science (n=216)

WanFang (n=47)

CNKI (n=48)

**Identification**

Records screened (n =670)

Records excluded based on the title and abstract (n =597)

Reports sought for retrieval

(n =73)

Reports not retrieved (n =12)

**Screening**

Reports assessed for eligibility

(n =61)

Reports excluded:

Non RCT(n=1)

Ineligible intervention(n=5)

Case study(n=10)

Outcome measures mismatch(n=12)

Lack of control group(n=6)

Inability to extract data(n=15)

Studies included in finally(n =12)

**Included**

*Consider, if feasible to do so, reporting the number of records identified from each database or register searched (rather than the total number across all databases/registers).

**If automation tools were used, indicate how many records were excluded by a human and how many were excluded by automation tools.

Source: Page MJ, et al. BMJ 2021;372:n71. doi: 10.1136/bmj.n71.

This work is licensed under CC BY 4.0. To view a copy of this license, visit <https://creativecommons.org/licenses/by/4.0/>
